# Contribution of Sex Differences to the Development of Cardiovascular Disease in Metabolic-Associated Steatotic Liver Disease (MASLD)

**DOI:** 10.3390/ijtm4040052

**Published:** 2024-12-09

**Authors:** Lucy C. Taylor, Gertrude Arthur, Marcella de Carvalho Cruz, David E. Stec, Olufunto O. Badmus

**Affiliations:** 1 Department of Physiology & Biophysics, Cardiovascular-Renal Research Center, University of Mississippi Medical Center, 2500 North State Street, Jackson, Mississippi 39216

**Keywords:** fatty liver disease, sex differences, estrogen, sexual dimorphism

## Abstract

Sex differences are a complex and crucial variable in developing and progressing metabolic and cardiovascular disease pathophysiology and clinical outcomes. The female sex, compared to the male sex, is protected from metabolic disturbances and their resulting cardiovascular events. However, the peculiar life phases associated with females, such as puberty, pregnancy, premenopausal, and menopausal stages, are all associated with different risks for the development of cardiovascular disease (CVD). Metabolic dysfunction-associated steatotic liver disease (MASLD), a condition of hepatic steatosis, and at least one feature of metabolic syndrome is associated with an increased risk of cardiovascular events. The risk of MASLD and its progression to the development of CVD differs between men and women. Differences in several factors, including formyl peptide receptor (FPR) 2, adipose tissue distribution, liver pyruvate kinase (LPK), and ketone body production, may underlie the sex differences in the risk of development of MASLD-induced CVD. Understanding the specific risk factors involved in the development and progression of MASLD between the sexes is crucial. This knowledge will provide important insights into the mechanisms responsible for its cardiovascular complications and can potentially lead to therapeutics targeted explicitly for each sex, offering new hope in the fight against MASLD-induced CVD.

## 1. Introduction

Metabolic dysfunction-associated steatotic liver disease (MASLD) is the latest nomenclature for hepatic lipid accumulation associated with metabolic syndrome [1]. Hepatic lipid accumulation of over 5% has shown a strong connection with metabolic disturbances such as dyslipidemia, obesity, hyperglycemia, insulin resistance, hypertension, and abnormal liver function tests [2]. To this end, a call for the review and renaming of non-alcoholic fatty liver disease (NAFLD) to metabolic dysfunction-associated steatotic liver disease was recommended [3]. During the last decade, we have seen a rise in the emergence of MASLD due to increases in metabolic dysfunction, such as obesity and type 2 diabetes [4,5], making MASLD a significant global public health burden. Presently, the global prevalence of MASLD is 30% [6], and it has been projected that over half of the adult population will have MASLD by 2040 [7]. Recently, there has been an increased interest in studying MASLD as a significant independent risk factor for cardiovascular disease (CVD) [8,9]. Convincing evidence shows that MASLD increases the risk of CVD morbidity and mortality even in the absence of traditional risk factors [10,11]. Cardiovascular disease is predominantly the cause of death among MASLD patients, as it has been revealed that more than half of MASLD patients die from cardiovascular complications [12,13]. Although the exact underlying mechanisms linking MASLD and CVD are not clear, possible mechanisms that have been implicated include insulin resistance/hyperglycemia, inflammation, dyslipidemia, oxidative stress, obesity, alteration in gut microbiota, and hepatokines [8,14]. Studies have also shown that cardiovascular events that have been observed in MASLD patients include atherosclerosis, hypertension, atrial fibrillation, increased intima-media thickness, and decreased flow-mediated dilatation indicating endothelial dysfunction, increased arterial stiffness, systolic and diastolic heart failure [8,15]. The European Association for the Study of the Liver (EASL), the American Association for the Study of Liver Diseases, European Society of Cardiologists, European Association for the Study of Diabetes (EASD), European Association for the Study of Obesity (EASO), and American Heart Association have all strongly recommended the integration of CVD risk assessments in all patients with MASLD [16–18]. Presently, there is no FDA-approved pharmacological treatment for MASLD, or for CVDs in patients with MASLD [19]. As of recent, the joint EASL-EASD-EASO provided guideline for the treatment of MASLD, and this includes lifestyle modification (such as weight loss, dietary changes, increased physical activities, and reduction in alcohol consumption), use of incretin-based therapies (semaglutide, tirzepatide) and bariatric surgery [16]. However, in MASLD patients with CVD, the current treatment strategy is based on weight loss and treatment of associated CVD risk factors involving the use of lipid-lowering agents, smoking cessation, treatment of hypertension, and glycemic control in patients with diabetes [20].

It is important to note that in human and experimental studies, female sex is protected from dysmetabolism and its associated cardiovascular consequences of MASLD. The incidence of MASLD and its increased risk of developing CVD more often affects men, whereas premenopausal women are equally protected from developing MASLD as they are from CVD [21,22]. Growing evidence suggests that there may be sex differences in the susceptibility to develop CVD induced by MASLD. However, the specific mechanisms by which female sex is protected from MASLD-induced CVD are unknown.

In the present review, we provide an overview of the literature on (A) the distinct role of sex differences on the development of MASLD and CVD, (B) the causative factors of sex differences in MASLD-induced CVD, (C) how sex differences influence the potential mechanisms of MASLD-induced CVD.

## 2. Prevalence of sex differences in the development of MASLD and CVD

Sexual dimorphism is an important variable that influences and plays a crucial role in MASLD development. Significant biological disparities exist between males and females due to differences in the chromosomal makeup and age-related fluctuations in sex hormone levels, and these are substantial contributory factors that influence metabolic disturbances. Previous pre-clinical and human studies have reported that males have a higher prevalence and severity of MASLD than pre-menopausal females [23]. Men are more prone to develop all the different stages of MASLD, including simple hepatic steatosis, metabolic dysfunction–associated steatohepatitis (MASH), fibrosis, cirrhosis, and hepatocellular carcinoma compared to women [24]. In a population-based study, Balakrishnan et al. reported that women had a 19% lower risk of hepatic steatosis than men in the general population [25]. Also, the metabolic effects of short-term fructose overfeeding (hypertriglyceridemia, hepatic insulin resistance, and elevated alanine aminotransferase) were attenuated in young females compared to males. [26]. A population study reported a higher prevalence of NAFLD in men (20.2%) than in women (15.8%) after adjusting for common risk factors [27]. In a community-based cohort study, Long et al. reported women had a reduced incidence of developing hepatic steatosis because of lower plasma triglyceride levels. [28]. Among younger patients, the prevalence of MASH showed an increase in the male population of less than 50 years of age, but the prevalence is higher in females of more than 50 years old, therefore associating sex differences in the incidence of MASLD with age differences [29].

Likewise, most experimental studies reported that MASLD is more severe in male experimental animals than females, irrespective of animal strain or genetic modification. In male and female mice fed a high-fat, high-sucrose diet, male mice exhibited higher levels of hepatic triglycerides that could be related to differences in body fat distribution and genetic regulation [30]. In an earlier study from our lab, increased hepatic lipid accumulation associated with hypertension was observed in male hepatocyte-specific peroxisome proliferator-activated receptor knockout mice but not in females [31]. In western diet-fed farnesoid X receptor knockout mice, males had more severe steatohepatitis than females [32]. In addition, a study using different male and female animal strains (Wistar, Long-Evans, and Sprague-Dawley rats, and C57/BL6 mice) revealed that males exhibit more significant hepatic steatosis on methionine choline-deficient diet than females [33]. In contrast to human studies, female C57BL/6J mice fed non-alcoholic steatohepatitis (NASH) diet exhibit reduced bile acid metabolism and enrichment in pathways associated with adhesion in stellate cells compared to male mice [34]. This data demonstrates the important role of sex differences in the increased susceptibility and severity of MASLD.

The occurrence of MASLD shares common risk factors with CVD that exhibit sex dependence. Metabolic syndrome (hypertension, dyslipidemia, hyperglycemia, obesity, and inflammation) remains a central factor in the development of both MASLD and CVD [35]. However, the risk of developing MASLD and its progression to the development of cardiovascular disease CVD may differ between males and females. Overwhelming clinical and experimental data have shown that incidence of both MASLD and CVD are more common in males than premenopausal females [36]. Hence, the interplay of hormones and sex-specific risk factors might contribute to female cardioprotection. However, it is essential to note that both males and females who exhibit MASLD possess a similar risk of developing CVD. This implies that women with pre-existing MASLD lose the protective effect that age-matched women without MASLD have against the development of CVD [37].

## 3. Factors contributing to sex differences in MASLD-induced CVD

Sexual dimorphism in the development and severity of both MASLD and CVD can be attributed to the type and levels of sex hormones, which can be influenced by age, reproductive status, lifestyle, ethnicity and genetic predispositions [21,38,39].

### 3.1. Estrogen and the estrogen receptor

**Figure 1.**
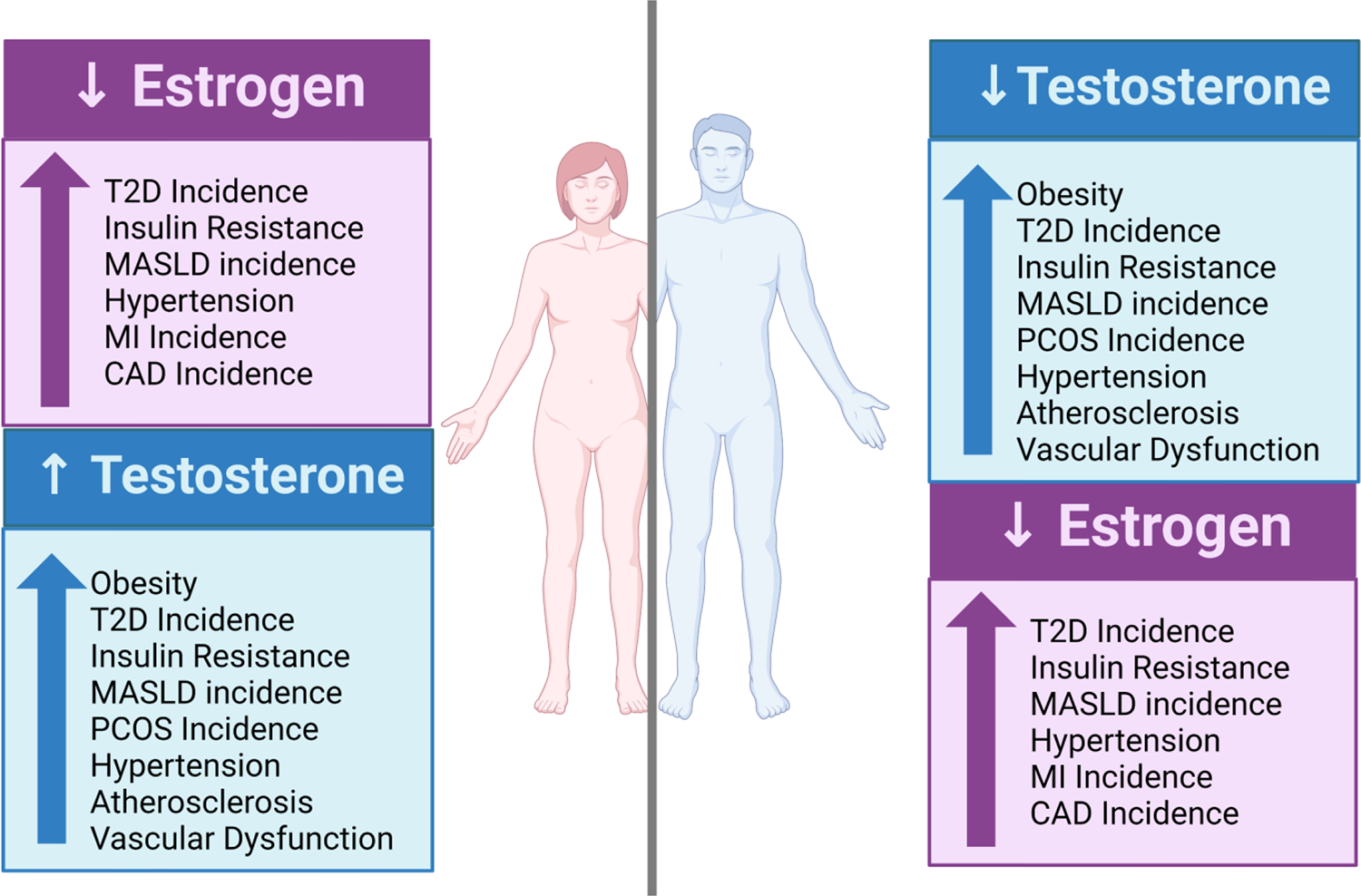
Impact of sex hormones on the development of MASLD and CVD. CAD, coronary artery disease; MASLD, metabolic-associated steatotic liver disease; PCOS, polycystic ovary syndrome; T2D, type 2 diabetes. Figure created with Biorender.com.

Estrogen, a predominant female sex hormone produced by the ovaries in premenopausal women, plays a vital role in inhibiting the development and progression of MASLD and CVD compared to their age-matched male (Figure 1) [36,40]. Endogenous estrogen serves as the primary female sex hormone, assisting in the development of female reproductive organs and participating in menstrual cycle regulation in premenopausal women. [41]. Beyond its role in reproduction, studies indicate its importance in regulating and improving metabolism and cardiovascular function. Recent studies have highlighted the potential protective role of estrogen against MASLD [42–45] and various cardiovascular diseases in both males and females [46]. Estrogen reduces MASLD directly through anti-steatotic and anti-inflammatory effects in hepatocytes and exerting anti-fibrotic effects in hepatic stellate cells [47]. Estrogen can also indirectly prevent MASLD by attenuating dyslipidemia, which contributes to the pathogenesis of MASLD [48]. Likewise, estrogen and estrogen receptors exert cardioprotective effects against atherosclerosis, ischemia/reperfusion injury on the myocardium, and increased arterial vasodilation [49,50]. More importantly, potential mechanisms that have been reported to link MASLD to CVD, such as reactive oxygen species, atherogenic dyslipidemia, systemic and vascular inflammation, and endothelial dysfunction, are inhibited by estrogen in premenopausal women [48,51].

Estrogen receptor-alpha (ER-α) activation by estrogen benefits lipid metabolism at the systemic level and in hepatocytes [24]. In 2000, Nemoto et al. demonstrated that aromatase-deficient mice unable to produce estrogen exhibit impaired gene expression and enzyme activities involved in peroxisomal and mitochondrial fatty acid β-oxidation, resulting in hepatic steatosis [44]. This study further showed that substituting estradiol in this model reduced hepatic steatosis and returned mitochondrial and peroxisomal fatty acid β-oxidation to levels seen in control mice [44]. Tamoxifen, a potent estrogen receptor antagonist commonly used in breast cancer treatment, has been shown to cause hepatic lipid accumulation in patients by increasing de novo fatty acid synthesis and inhibiting the mitochondrial fatty acid β-oxidation in the hepatocytes [52]. In mice lacking peroxisome proliferator-activated receptor- α (PPARα), a nuclear receptor involved in lipid metabolism, hepatic and cardiac lipid accumulation was observed in addition to death in 100% of males but only 25% of females. In the same study, two weeks of pretreatment with β-estradiol in males rescued them from the cardiometabolic phenotype, suggesting the involvement of estrogen in hepatic and cardiac lipid accumulation [53]. In another experimental study, a model of postmenopausal hypertension using ovariectomized Dahl salt-sensitive rats fed a low-salt diet, hypertension correlated with increased type 1 angiotensin receptor levels as compared to control age-matched Dahl salt-sensitive rats fed a low-salt diet [46]. In addition, estrogen therapy attenuates hypertension and activity of the renin-angiotensin system in female ovariectomized Dahl salt-sensitive rats fed a low-salt diet [46]. Furthermore, a study by Heine et al. revealed that eliminating estrogen receptor alpha (ERα) in mice leads to heightened white adipose tissue accumulation, insulin resistance, and glucose intolerance in both male and female mice [54]. Estrogen replacement therapy attenuates the risk of MASLD and CVD in menopausal women to premenopausal levels [48,49]. These findings suggest that estrogen in premenopausal females exerts hepato-cardio protection, and endogenous or exogenous estrogen can confer protective benefits against MASLD and CVDs in both male and female subjects.

### 3.2 Progesterone

Progesterone is another predominant female hormone that protects hepatocytes and the cardiovascular system [55,56]. Even though men have lower progesterone levels than postmenopausal women, progesterone in males aids reproduction and benefits overall health. Several studies have documented the protective effects of progesterone on hepatic and cardiovascular functions in animal models. For instance, progesterone treatment of male rodents with hepatic fibrosis attenuated the development of liver fibrosis and inflammation [55]. Protective actions of progesterone in the cardiovascular system include lowering blood pressure, inhibiting coronary hyperactivity, blood vessel vasodilation, and increased sodium excretion [57,58]. Dhote and Balaraman, reported that the cardioprotective effect of progesterone on myocardial ischemia/reperfusion injury is sex- and age-specific. They observed that progesterone administration significantly reduced infarct area, lipid peroxidation, creatine kinase activity, ventricular tachycardia, and increased glutathione levels in female rodents with ischemia/reperfusion injury; however, these cardioprotective effects of progesterone were lost in males with ischemia/reperfusion injury as well as ovariectomized females with ischemia/reperfusion injury [56]. This suggests a cardioprotective interaction between progesterone and estrogen in premenopausal females. The importance of estrogen is suggested by the fact that increased progesterone is corrected for ischemia/reperfusion cardiac injury during premenopausal but not in males and females with postmenopausal status.

Progesterone hormone inhibits agonist-induced vasoconstriction in vascular tissues by mediating calcium channel activity [57]. Also, progesterone influences membrane progesterone receptor-α to rapidly activate second messenger pathways in vascular endothelial cells, causing an increase in nitric oxide that exerts cardiovascular protection [58]. Since females naturally have more progesterone than males, this might contribute to women’s hepatic and cardiovascular protection. However, large-scale human studies in both sexes are needed to further evaluate the potential role of sex on the impact of progesterone on MASLD and CVDs.

### 3.3 Androgens

Androgens (testosterone, dihydrotestosterone, and dihydrotestosterone) are predominantly hormones associated with male sexual development; however, androgens also play significant roles in physiological processes in women. Recent studies have linked androgen imbalances with the development or progression of MASLD and various cardiovascular diseases in women [59,60]. It has been established that low serum testosterone levels in males are associated with metabolic disturbances (insulin resistance, obesity, hyperglycemia) and the development of CVDs (Figure 1) [61]. Androgen deficiency has been linked to hepatic steatosis in men, indicating that normal androgen levels prevent the development of hepatic steatosis [60]. Studies have demonstrated that decreased levels of fasting serum and free testosterone in men are associated with hypertension, myocardial infarction, and coronary artery disease [62,63]. Testosterone deficiency in men is also linked to insulin resistance and increased visceral fat accumulation. Both of these conditions are central to metabolic syndrome and the development of CVDs and are attenuated with testosterone replacement [64]. Conversely, elevated androgen levels in women have been observed in hepatic steatosis associated with polycystic ovary syndrome (PCOS) (55), as well as increased visceral adipose accumulation and insulin resistance [64]. PCOS, characterized by elevated androgen levels, chronic anovulation, and polycystic ovaries, remains a prevalent endocrine disorder among women of reproductive age [65]. More than half of women with PCOS have MASLD, and these patients clearly possess a higher risk of developing CVDs [42,59,65]. The pathophysiology of PCOS involving insulin resistance can be a driver of cardiometabolic diseases such as MASLD and CVD [66]. A model of PCOS induced in rodents by dihydrotestosterone treatment results in insulin resistance and hepatic steatosis associated with impaired mitochondrial function, apoptosis, and autophagy [59]. Women with PCOS also have an increased prevalence of CVD precursors such as hypertension, coronary artery calcification, atherosclerosis, and vascular dysfunction [67–69]. These findings suggest that an increase in circulating androgen in males is beneficial for hepato-cardio function (Figure 1). However, androgen increase in women is associated with metabolic disturbances, including MASLD, and an increased risk for the development of CVD.

### 3.4. Insulin-like growth factor-1 (IGF-1)/ growth hormone (GH)– axis

The growth hormone (GH)/ insulin-like growth factor-1 (IGF-1)– axis is a key endocrine system that regulates metabolism in humans [70]. In the liver, GH is the major stimulator of IGF-1, and the downstream effects of IGF-1 include reducing visceral fat, lipogenesis, triglyceride accumulation, and improving insulin resistance to inhibit the development of MASLD [71]. Hence, this axis is often found to be dysregulated in the condition of MASLD, with MASLD patients experiencing lower levels of both GH and IGF-1, which can contribute to the progression of the disease [72]. Interestingly, the GH/IGF-1 axis is significantly impacted by sex hormones, thereby exhibiting a sexual dimorphism in MASLD severity. In females, estrogens interact with estrogen receptors to reduce the sensitivity of the liver to GH, thereby decreasing the production of IGF-1 and acting as an inhibitor of the axis [71]. Lower IGF-1 levels associated with high estrogen can potentially contribute to the development and progression of MASLD by impairing liver function and promoting hepatic lipid accumulation [73]. In males, testosterone stimulates GH secretion and consistently elevates total IGF-I concentrations [74]. Hence, during MASLD, higher IGF-1 and more favorable fibrosis scores were seen in men but not women [75]. This may contribute to sex differences observed in MASLD and MASH since women with MASLD are more likely to have an increase in disease progression.

### 3.5 Adipokines

Adipokines are a group of molecules produced from adipocytes, and they function in regulating appetite, blood pressure, inflammation, immune response, and metabolism [76]. Aberration in adipokine production plays a critical role in the development of CVDs mediated by MASLD. According to existing research, adiponectin is a type of adipokine that increases insulin sensitivity, lowers inflammation and possesses anti-atherogenic, anti-obesogenic, cardiac, and hepato-protective properties [77,78]. Adiponectin exerts its beneficial effects by binding to AdipoR1 and AdipoR2 receptors in the liver to activate the AMPK pathway, suppress hepatic gluconeogenesis and de novo lipogenesis, decrease the expression of SREBP-1 and activate PPAR-α that enhance fatty acid oxidation to prevent MASLD [77,79]. Decreased levels of adiponectin in the bloodstream of patients with MASLD correlate with an increased risk of developing CVD [80]. Adiponectin levels decrease up to 20–40% during MASLD [81], and low adiponectin levels significantly elevate the risk of adverse cardiovascular events through mechanisms like insulin insensitivity, inflammation, atherosclerosis, enhancement of monocyte adhesion to endothelial cells, and obesity [78,81]. Adiponectin production is higher in females than males and might contribute to protecting the hepato-cardiac axis in females. Sex differences in adiponectin production start at puberty when there is a rapid decline in adiponectin production in boys, which could be attributed to changes in fat mass distribution and testosterone levels. Previous human and animal studies revealed that adiponectin decline was inversely related to testosterone levels in adolescents [82], and androgen receptor null male mice exhibit insulin sensitivity and increased adiponectin levels [83]. Furthermore, estradiol hormone stimulates adiponectin expression both *in vivo* and *in vitro* [84], while in postmenopausal women, decreased production of estrogen and sex-hormone binding globulin with a concurrent rise in free testosterone are connected with decreased production of adiponectin [85]. This evidence implies that therapeutic interventions that increase circulating adiponectin may be necessary for improving MASLD and its associated complications of CVDs.

In patients with MASLD or MASH, changes in adiponectin and leptin levels, another type of adipokine, are observed [86]. While adiponectin levels decline in MASLD, leptin levels are elevated [87]. Leptin can act directly via the leptin receptors in the liver or indirectly via the central nervous system mechanisms [88]. Leptin exerts a protective role against MASLD by increasing insulin sensitivity and lipid oxidation and reducing hepatic lipid accumulation [86,89]. However, studies revealed that when circulating leptin is elevated, it contributes to the development of MASLD, and it is also linked to inflammation, angiogenesis, and fibrogenic processes that aid the progression of MASLD to MASH [89]. Elevated leptin levels associated with MASLD may drive the increased risk of CVDs. Elevated leptin levels promote high blood pressure, platelet aggregation, inflammation, insulin resistance, and endothelial dysfunction [90–92]. In addition, increased leptin observed in obesity may contribute to the heightened risk of CVDs in these patients [93]. Leptin has been reported to be expressed differently in men and women [94]. Women have higher leptin levels irrespective of postmenopausal status due to an increased amount of adipose tissue and an increased production rate of leptin per unit mass of adipose tissue [95,96]. Leptin’s role in MASLD-induced CVDs may be dependent on sex factors, which can influence their cardiometabolism differently. For example, a human study showed an association between leptin gene variants and hypertension in postmenopausal women and a link between increased plasma leptin and hypertension in premenopausal women compared to men [97]. Furthermore, obesity-induced leptin resistance confers a higher risk of CVDs in women than men [98]. However, in animal studies, high-fat diet fed aged female mice showed an increase in the adiponectin/leptin ratio that protected the female from obesity and its associated cardiovascular events compared to males of the same age [99]. This evidence, taken together, may explain sex differences in the activity of leptin-induced cardiometabolic disturbances, especially in females.

### 3.6 Plasma bilirubin levels

It has recently been established that bilirubin acts as a signaling hormone to attenuate hepatic lipid accumulation [100–103]. Higher levels of plasma bilirubin have been associated with a lower risk of CVD due to its ability to activate PPARα and its antioxidant, anti-inflammatory, and platelet-inhibitory actions [104–106]. However, sex differences in plasma bilirubin levels remain controversial. Under normal conditions, average bilirubin levels are higher in males than in females [101]. While some studies reported that increased serum total bilirubin levels are associated with a decreased risk of MASLD and CVD in both men and women [107,108], few studies observed sex differences in the relationship between bilirubin levels and MASLD-induced CVD [109]. For example, an earlier study using both humans and animals showed that estrogen–estrogen receptor α signaling enhances bilirubin metabolism that supports liver regeneration in females more than males; this might contribute to shorter liver function recovery usually observed in females than in males [110]. Toth et al. reported a 2-fold increase in hepatic expression and activity of heme oxygenase in females compared to males [111]. Heme oxygenase is the enzyme that degrades heme to produce biliverdin, which is immediately reduced to bilirubin. Bilirubin binds to PPARα and enhances its activity to inhibit hepatic lipid accumulation [106]. A recent longitudinal study revealed that increased serum total bilirubin levels decreased the incidence rate of metabolic syndrome in women but not men [109]. Zhang et al. found elevated serum bilirubin to reduce the risk of coronary heart disease in postmenopausal females [112]. On the contrary, a genome-wide association study reported that bilirubin’s protective and antioxidant effect may be higher in men than women [113]. Due to these conflicting results, more animal and clinical studies are needed to ascertain the potential role of sex differences in the relationship between bilirubin hormone and the incidence of MASLD and CVD.

## 4. Reproductive Status

**Figure 2.**
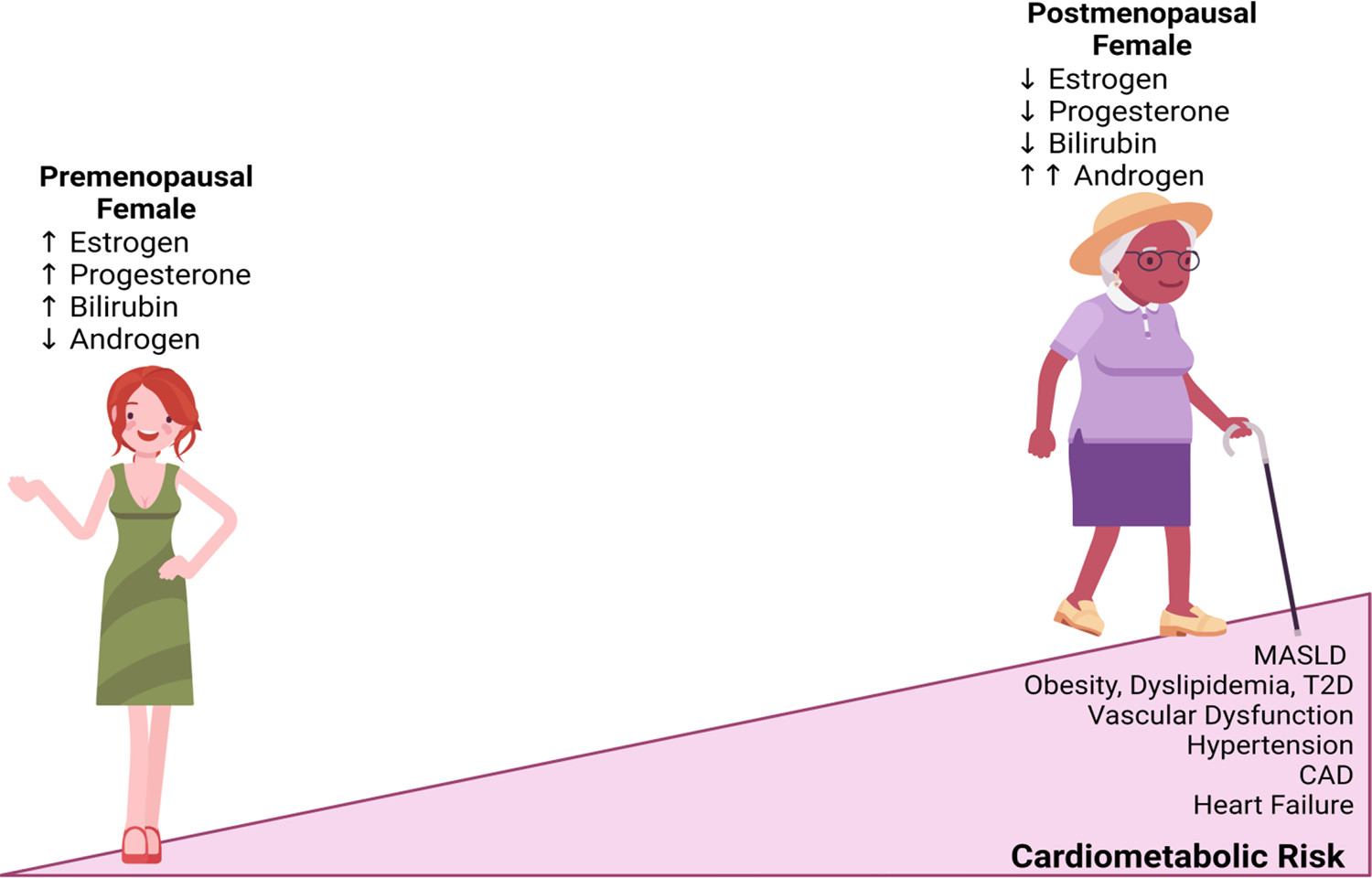
The decline in female cardioprotective sex hormones increases the risk of cardiometabolic disorders. CAD, coronary artery disease; MASLD, metabolic-associated steatotic liver disease; T2D, type 2 diabetes. Figure created with Biorender.com.

Reproductive status such as puberty, pregnancy, premenopausal, and menopausal phases potentially impact the development and severity of MASLD and CVD (Figure 2) [114,115]. The prevalence of MASLD in children and adolescents has significantly increased due to the incidence of obesity, although there are instances of MASLD in non-obese. The global prevalence of MASLD in children is between 5% - 10% and differs by sex, as girls have a prevalence rate of 7.9% compared to 11.1% in boys with normal body mass index [116]. Similarly, in children with obesity, the prevalence of MASLD was also higher in boys, with a prevalence rate of 29.4% and 22.6% in girls [117,118]. In a large cross-sectional study on obese children and adolescents, the occurrence of MASLD is more predominant in boys than in girls [119], suggesting that the sex differences in the propensity to develop MASLD exist at an early age and extend into adulthood. Pregnancy is a crucial period for cardiometabolic health in women, as physiological changes during pregnancy influence fat deposition in the liver and can promote CVD. Furthermore, studies have shown that pregnancy in itself is a significant stressor on the cardiovascular system, which can promote the development of CVD later in life [120]. Presently, there is a rising trend of obesity and insulin resistance in women of childbearing age [121], putting them at risk of developing MASLD-induced cardiovascular complications. A woman can have a preexisting condition of MASLD before pregnancy or develop the condition during pregnancy. The occurrence of MASLD in pregnancy has significantly increased in the last decade, and it is associated with adverse cardiovascular outcomes such as hypertension, eclampsia, and gestational diabetes mellitus [122,123]. A study recently revealed that the prevalence of MASLD in pregnant women is around 14%–18% [124]. Moreover, any stage of fatty liver disease, from simple steatosis to MASLD, is an independent risk factor that increases the risk of cardiovascular events during pregnancy and delivery [121,125]. Developing MASLD-induced CVD in pregnancy may result from physiological and pathological alterations in hormone levels, increased body fluid volume, and rapid weight change during pregnancy [120,122]. The findings that metabolic changes in the liver during pregnancy influence cardiovascular complications place pregnant women at higher risk of MASLD-induced CVDs compared to their age-matched male counterpart. Hence, there is a need for pregnant women to be screened for MASLD before pregnancy. Also, prevention or therapeutic intervention for CVDs in pregnant women with MASLD is essential.

Due to the protective effects of estrogen discussed earlier in this article, premenopausal women are protected mainly from MASLD and its associated cardiovascular diseases, such as coronary artery disease, arterial hypertension, diabetes, obesity, and dyslipidemia (Figure 2) [126]. Clinical study shows that ovulatory abnormalities and estrogen deficiency in premenopausal women promote MASLD as well as atherosclerosis and increase the risk of heart failure, suggesting that female sex hormones protect the cardiovascular system of premenopausal women [42,43,127,128]. However, it has been observed that premenopausal women with MASLD lose cardiovascular protection and have a similar risk of developing CVD as men with MASLD; this implies that the cardioprotective effect of estrogen is likely lost upon the development of MASLD in premenopausal women [37]. It is important to note that premenopausal women use hormonal contraception for contraceptive and non-contraceptive reasons. Premenopausal women with MASLD on hormonal contraception exhibit more severe hepatic injury and inflammation than their male counterparts [129]. However, while some studies reported a lower risk of CVD events in young women using exogenous hormones, some studies reported an increased risk of cardiovascular events in premenopausal women [130,131]. The benefits or risks of contraceptive use can be attributed to the type of contraception. For example, estrogen-containing methods such as combined oral contraceptives in young women increase the risk of myocardial infarction, venous thromboembolism, and ischemic stroke [132,133]. On the other hand, progestogen-only contraceptives are associated with substantially less risk of cardiovascular events than combined oral contraceptives [134]. There is a dearth of information about whether the route of contraceptive administration (oral, transdermal, or vaginal) increases cardiovascular risk. Hence, more studies are needed to investigate this area and the type of contraception should be considered in premenopausal women, especially in those with existing cardiovascular risk factors like MASLD.

During menopause, there is a sharp increase in the occurrence of MASLD and CVDs. The risk of MASLD and the development of CVD in women escalates following menopause, which supports the idea that estrogen has protective effects against cardiometabolic diseases in premenopausal women [42,114]. Results from a cross-sectional study by Gutierrez-Grobe et al. revealed that postmenopausal have a greater prevalence of MASLD than premenopausal women, and postmenopausal participants diagnosed with PCOS had the highest incidence of MASLD. In the same study, higher serum estradiol concentrations were found in premenopausal women without MASLD compared to low estradiol levels in postmenopausal women with MASLD [42]. Due to a decline in estrogen levels, postmenopausal women begin to show increased insulin resistance and visceral abdominal fat, leading to an increased incidence of MASLD and CVDs [135]. Additionally, the risk of developing hypertension, diabetes mellitus, and metabolic syndrome increases after menopause, specifically in postmenopausal women with MASLD [114]. Post-hormonal treatment, also known as hormone replacement therapy, is used to replace the diminishing levels of female sex hormones associated with the menopausal phase. The two main types of post-hormonal treatment (estrogen therapy only or combination therapy of estrogen and progesterone) lessen postmenopausal symptoms such as mood swings, hot flashes, night sweats, osteoporosis, and vaginal dryness [136]. Several observational studies have shown that postmenopausal women receiving hormone replacement therapy have a reduced rate of developing MASLD and cardiovascular mortality compared to women who do not receive hormones [137–139]. On the contrary, results from randomized clinical trials reported that hormone replacement therapy increases the risk and events of CVD in postmenopausal women [140,141]. The reasons for this contradictory role are unclear, although the timing and type of post-hormonal treatment may play significant roles. For example, hormone replacement therapy initiated in women younger than 60 years old or women near menopause confers cardiovascular benefits. At the same time, an increased risk of heart disease, venous thromboembolic events, and pulmonary embolism were reported in women who started hormone replacement therapy after more than 10 years of menopause [139]. Also, an increased risk of uterine cancer is associated with estrogen use only without progestin [142]. All these taken together, the type of hormone replacement therapy and time of initiation should be put into consideration in menopausal women to prevent cardiovascular events.

## 5. Age

Research suggests that age-related differences play a significant role in developing MASLD and various CVDs in males and females. MASLD is a disease that progresses and worsens over time with aging [143]. As people age, the risks associated with metabolic and cardiovascular disease tend to escalate in both males and females. Men exhibit more endothelial dysfunction and arterial stiffness than women across all age groups. However, after the sixth decade, women experience a faster progression of vascular dysfunction [144]. Although some studies suggest that the decline in cardiovascular health in women is associated with the onset of menopause and a disrupted estrogen-androgen balance [143,144], in contrast, others relate this decline in cardiovascular health to general aging [145]. An earlier cohort study showed that men with MASLD are at risk of developing incident CVD falls at age 50–64, while women with MASLD were at greater risk of developing CVD at age 65–74 [144]. These findings show that males develop MASLD and have an increased risk of cardiovascular events at a younger age as compared to females. A separate study that investigated age and sex differences in cardiovascular mortality in MASLD population reported that cardiovascular mortality rate in men was significantly higher (12.4%) than that observed in women (7.7%); however, this study also found that females with MASLD less than 60 years had a higher risk of heart-related death than males of similar age [145]. However, these results are not consistent with findings from a national study in the United States that revealed a fourfold increase in mortality among both men and women diagnosed with MASLD between the ages of 45 and 54 and another study that reported the risk of mortality was similar between individuals without MASLD and those diagnosed between the ages of 55 and 84 [146]. These results are further supported by Kagansky et al., who reported no significant differences in metabolic abnormalities between octogenarians with MASLD and those without, suggesting that MASLD is more or less a benign condition in older adults [147]. More studies are needed to determine the role of MASLD in promoting CVD in populations of older adults more than 80 years old.

## 6. Race and ethnicity

There is an increase in the prevalence of MASLD and its associated CVD risk factors over time across all race/ethnic groups [148,149]. According to existing research, racial/ethnic disparities may influence sex differences in the cardiovascular outcome of MASLD [148]. Earlier studies within the United States have established that the population of Hispanic descents have the highest prevalence of MASLD, and Mexicans further show the greatest burden of MASLD among the Hispanic population [150]. This observation was consistent with another population-based cohort study that found Hispanics had about twofold higher prevalence of hepatic steatosis (45%) compared to Blacks (24%) and roughly 1.4-fold higher prevalence compared to Whites [151]. Some of the explanations given for the high incidence of MASLD within the Hispanic population include genetics, environmental exposures, health behaviors, and socioeconomic disparities [149]. Studies have also shown that in addition to the Hispanic population, individuals with MASLD from African and Native American/Alaskan ancestry have an increased risk of CVDs, diabetes mellitus, liver-related events, and mortality rate than patients of European ancestry [152]. Recently, a cohort study showed that the severity of metabolic syndrome in individuals with MASLD is higher in Mexican-American and Black non-Hispanic females than White non-Hispanic females, but black non-Hispanic males had lower severity of metabolic syndrome than White non-Hispanic males [153].

Cardiovascular outcomes in individuals with MASLD reveal differences across sex and race/ethnicity. Males with MASLD who are Non-Hispanic Blacks have the highest risk of death from CVD, followed by males from non-Hispanic whites, then Mexican Americans. Interestingly, females with MASLD were at lower risk of CVD mortality across different races [148]. These findings taken together show that while the prevalence of MASLD and severity of metabolic syndrome is highest in the population of Mexican origin, Black non-Hispanic males with MASLD have the highest risk of cardiovascular events and mortality. Hence, clinical interventions that target the prevention and management of CVDs in susceptible populations with MASLD should be adopted.

## 7. Genetic predispositions

In 2008, the first gene variant, patatin-like phospholipase domain-containing protein 3 (PNPLA3) is implicated in hepatic lipid accumulation was discovered [154]. Genome-wide association meta-analysis has identified an association between MASLD and variants of the following genes: PNPLA3, TM6SF2, MBOAT7, HSD17B13, GCKR, TRIB1, MTTP, GPAM, MARC1, and APOE [154,155]. These earlier studies indicate that MASLD can be genetically influenced.

The I148M variant in the PNPLA3 gene is the most investigated because it accounts for the most significant fraction of variability and severity in steatohepatitis [156]. It is a variant with the most significant impact on the risk of developing progressive MASLD and the incidence of other metabolic risk factors and associated CVDs [157]. The Human PNPLA3-I148M variant increases the accumulation of polyunsaturated fatty acids within the liver and predisposes an individual to hepatic steatosis [158]. Even though men are more vulnerable to developing every stage of hepatic steatosis than females, studies have shown that women carrying the *PNPLA3-* I148M variant can be at greater risk of developing MASLD than men [159]. In an obesity study, hepatic PNPLA3 expression was higher in obese women than in obese men. The study further shows that PNPLA3 was induced by estrogen receptor-α agonists, suggesting a correlation between estrogen levels and expression of PNPLA3 in the liver [159]. In summary, the PNPLA3 I148M variant as a risk factor for MASLD is sex-dependent, and PNPLA3 I148M could influence the progression of MASLD and cardiovascular outcomes in female subjects.

## 8. Lifestyle

Unhealthy lifestyles, including smoking, an unhealthy diet, and a sedentary lifestyle, are critical factors that contribute to the pathophysiology of MASLD and CVDs. Studies have shown that men are likely to smoke tobacco every day and at higher rates than women [160]. Smoking could be an avoidable risk factor in the development and progression of MASLD and its complications of CVDs. Several studies have documented the independent role of cigarette smoking in the progression of fibrosis in MASLD [161–163]. Some of the mechanisms shown to be involved in tobacco use and the progression of fibrosis in MASLD include insulin resistance, proinflammatory cytokines, nicotine-activated collagen synthesis, hepatocyte iron accumulation, and activation of stellate cells by oxidative stress [161,163–166]. Furthermore, the mechanistic pathways by which smoking increases the risk of CVDs include endothelial damage, atherosclerosis, inflammation, thrombosis, cardiac tissue hypoxia, and platelet activation [167–170]. As of 2016, 22.8% of men in the United States smoked, compared to 18.3% of women [171]. Similarly, in a study in Thailand, 67.0 % of males and 41.9% of females are likely to smoke daily [160]. Recent findings in the United States revealed that although the prevalence of smoking is higher in men, women are likely to find it challenging to quit smoking compared to men, increasing the number of women who smoke over time [172]. The distribution of prevalence in smoking adults varies depending on the country’s status. For example, the prevalence of smoking in females is significantly reduced in developing countries (3.1%) compared to developed countries (17.2%), whereas the prevalence of smoking in males is similar [173]. These findings imply that increased smoking in men could be a preventable risk factor that is contributing to the increased prevalence of MASLD and CVDs observed in men. Lifestyle change through smoking cessation could be beneficial in attenuating the progression of CVDs induced by MASLD.

Sex could be a biological variable in diet-induced MASLD and CVDs. Excessive caloric intake is associated with obesity, type 2 diabetes, MASLD, and CVDs [174,175]. In animals and humans, females consume fewer calories per day than males [176,177]. Sex differences in eating behaviors are influenced by sex hormones [177]. For instance, estradiol, the primary female hormone, influences food intake via estrogen receptor-α (ERα) signaling [178] and other factors such as leptin, cholecystokinin, ghrelin, and insulin [178]. In human and animal studies, elevated estradiol level during ovulation is linked with decreased food intake [177,179]. This was further confirmed in experimental ovariectomized animals that had significantly increased food intake but, when given estradiol replacement, had decreased food intake [179]. On the contrary, orchiectomy in male adult rats led to decreased food intake, but exogenous androgen replacement restored increased food intake behaviors [180], suggesting that while female hormones attenuate food intake, male hormones promote increased food intake. However, further studies are needed to investigate the role of androgens in food intake and the mechanisms by which androgens influence food intake.

A sedentary lifestyle is an independent predictor of MASLD and CVDS, and this could be sex and age-dependent. In Canadian children and adolescents, girls are more sedentary than boys. Similarly, among adults of 35–49 years, women had more sedentary time than men [181]. However, in the United States, men aged 60 and above showed lack of physical activity and sedentary behavior more than women of their age [182]. Interestingly, women have greater gain of reduced risk of CVDs mortality with physical activity than men [183]. Since females are at greater risk of sedentary behavior, more efforts should be made to motivate women towards regular physical exercise.

## 4. Mechanistic factors potentially responsible for sex differences in MASLD-induced CVD

### 4.1 Formyl peptide receptor 2

**Figure 3.**
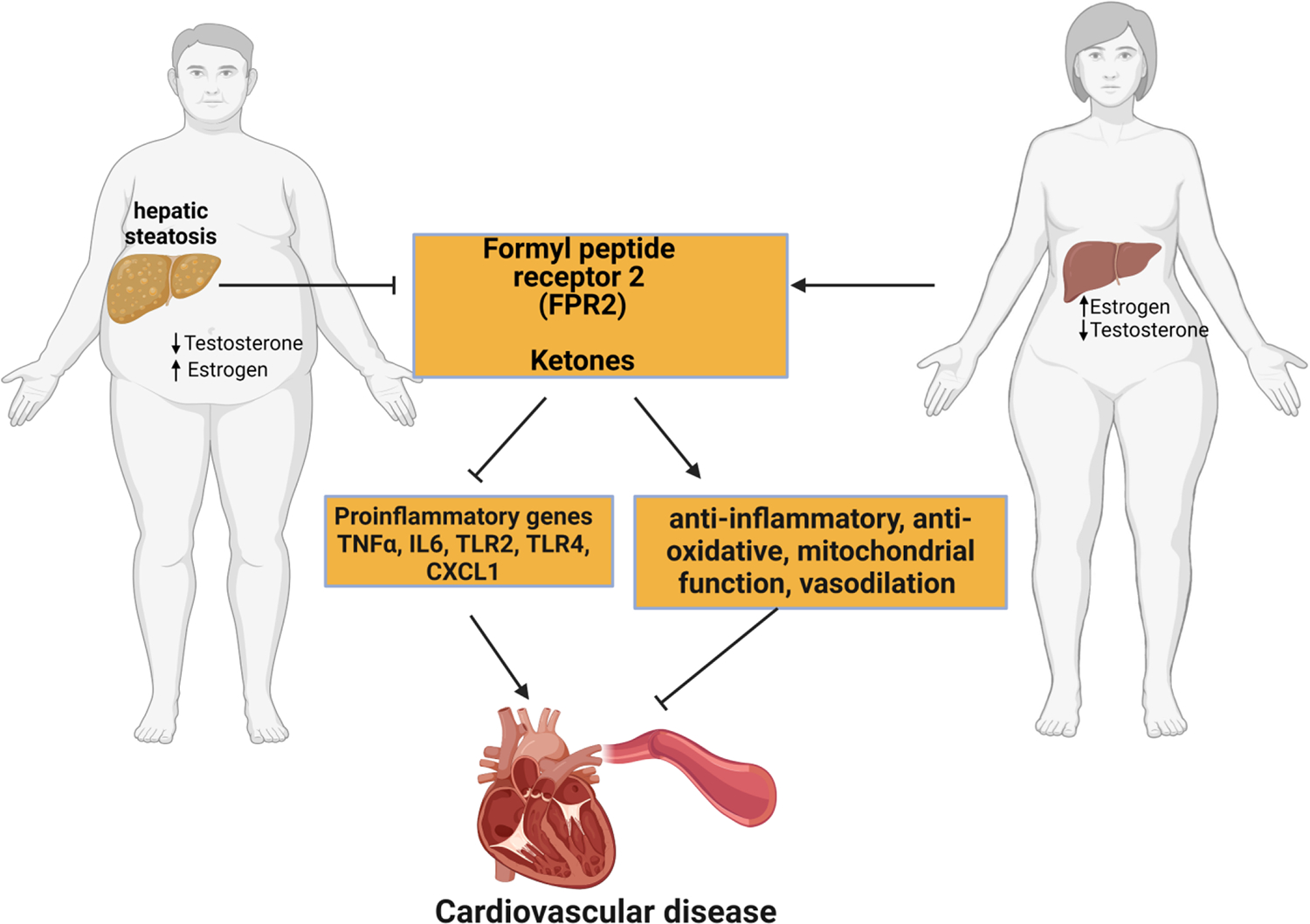
Potential sex difference mechanisms are responsible for the development of cardiovascular in MASLD. CXCL-1, Chemokine (C-X-C motif) ligand 1; IL6, Interleukin 6; TNFα, Tumor necrosis factor-alpha; TLR- Toll-like receptor. Figure created with Biorender.com.

Formyl peptide receptor (FPR) 2, which is part of the formyl peptide receptor family, including formyl peptide receptors 1 and 3 in humans, are transmembrane chemoattractant G-protein coupled receptors [184] that mediate both pro and anti-inflammatory responses due to damage-associated or pathogen-associated molecular patterns (Figure 3) [185]. Formyl peptide receptors are expressed in immune cells, particularly FPR1 and 2, while FPR3 is expressed in specific immune cells. Formyl peptide receptors, except FPR3, are expressed in hepatocytes and endothelial cells. FPR2 is also expressed in endothelial, astrocytoma, and neuroblastoma cells [186]. FPR2, depending on the peptide or lipid ligand, elicits either a pro- or anti-inflammatory response [187]. Thus, the binding of ligands such as lipoxin A4 and resolving D1 elicits an anti-inflammatory response [188] that protects from hepatic bacterial infection [189], hepatocyte death and mitochondrial dysfunction [190], alleviation of ischemia/reperfusion-induced liver injury through increased phagocytosis of dead and dying cells by macrophages [190] as well as enhanced apoptosis in cancerous liver cells [191]. However, other ligands, such as serum amyloid A, stimulate the proinflammatory action of FPR2, which exacerbates atherosclerosis [192] and diabetic retinopathy [193] by producing proinflammatory cytokines.

In MASLD, FPR2 has been shown to protect from lipotoxicity and suppress MASLD progression in mice fed a choline-deficient, L-amino acid-defined high-fat diet [194]. Furthermore, global deficiency of FPR2 in mice enhances inflammation and liver injury after lipopolysaccharides (LPS) treatment [195]. FPR2 loss also increases proinflammatory genes such as TNF-α, IL-6, TLR2, TLR4, and CXCL1 [196]. However, other findings show that loss of FPR2 alleviates hepatic steatosis in high-fat-fed mice [196]. It is worth noting that the conflicting findings may be due to the different genetic backgrounds of the mice used and their diets. While mice in the high-fat diet study showed MASLD through an increase in adipose tissue (discussed further in this review), mice in the choline-deficient, L-amino acid-defined high-fat diet study did not have a difference in adipose tissue, highlighting how different diets and models can affect the contribution of FPR2 to MASLD.

MASLD prevalence and severity are higher in men than in premenopausal women, who quickly catch up with males upon menopause [28,197]. Animal models also show this phenomenon, and FPR2 expression could contribute to this phenotype. Expression of FPR2 is higher in female mice with intact gonads and reduces after ovariectomy [194]. Along this line, female mice with intact gonads are protected from hepatic steatosis, whereas ovariectomized female mice develop hepatic steatosis and progress much more quickly in MASLD severity [194]. Moreover, inflammatory chemokines such as CCL2 and CXCL2 are increased in high-fat diet-induced MASLD in males and ovariectomized females [198], showing that female sex hormones protect against MASLD through mechanisms that include the FPR2 pathway in mice. Inflammation is a well-documented driver of cardiovascular disease. Thus, mechanisms that prevent or minimize global inflammation, such as FPR2, could benefit postmenopausal women and men by improving overall cardiovascular health.

Paradoxically, human studies show that FPR2 expression is increased in atherosclerosis in males but not females [199]. This finding has been recapitulated in mice, showing that disruption of FPR2 in ApoE^−/−^ mice attenuates atherosclerosis [192,200]. The driving factor in this disease condition is increased proinflammatory FPR2 ligand and serum amyloid A [192]. However, increased FRP2 expression protects from ischemia/reperfusion injury [201]. These results demonstrate that the role of FPR2 in health and disease is dependent on its ligand activity, which can be sex-dependent due to the varying expression of FPR2 in males and females (Figure 3).

### 4.2 Adipose tissue distribution

Obesity is a significant driver of MASLD development. Adipose tissue, particularly white adipose tissue (WAT), is a primary storage site for energy during the fed state and, in the fasted state, is a major source of triglycerides and free fatty acids [202]. These processes are highly controlled by hormones like insulin (in the fed state) and catecholamines (in the fasted state) [203]. By serving as the primary site for energy storage in triglycerides, the adipose tissue acts as a buffer to ensure whole-body energy homeostasis and prevent ectopic lipid deposition, which causes lipotoxicity [204]. Increased energy intake leads to a state of obesity that drastically increases adipose tissue volume. However, adipose tissue distribution is highly sex-dependent and has important implications for MASLD and other metabolic diseases [205] (Figure 3).

Adipose tissue is distributed into subcutaneous adipose tissue, 80–90% of total body fat, and visceral adipose tissue, 10–20% of total body fat [206]. Subcutaneous adipose tissue (SAT) comprises WAT in the subscapular, gluteal, abdominal, and femoral areas, while visceral adipose tissue (VAT) is WAT in the intrabdominal cavity [206,207]. Both rodent and human studies show that sex hormones affect adipose tissue distribution. Higher estrogen in females leads to a greater tendency to store WAT in gluteal and femoral areas, while higher testosterone in males shifts WAT storage to the visceral, intraabdominal area [208–210]. Moreover, sex hormone fluctuations lead to changes in body fat distribution. This is seen in postmenopausal women who have higher WAT accumulation in the VAT than premenopausal women [205]. Apart from SAT and VAT, other WAT storage includes the epicardial and perivascular sections [211].

Since SAT is more abundant and under the skin, WAT storage in the SAT could be the major buffer and storage site during excess energy intake to prevent metabolic dysfunction [205,206]. Also, because the SAT is under the skin and not in contact with other organs, metabolic fluctuations can be contained to a greater extent so as not to affect other organ systems [205,212]. However, VAT is in direct contact with major organs such as the liver and drains venous blood into the portal vein, bringing the liver into direct contact with free fatty acids, adipokines, and inflammatory mediators secreted by visceral adipocytes [212].

Moreover, adipose tissue releases adipokines, which modulate lipid storage and utilization, lipid dysregulation, liver steatosis, and cardiovascular dysfunction [213]. Adipokines such as leptin, adiponectin, visfatin, transforming growth factor beta, and interleukin-6, among others, play a significant role in energy utilization, storage, and inflammation that leads to metabolic dysfunction [214]. Interestingly, the expression and release of some adipokines are sex-dependent. Leptin, for example, is higher in women than weight-matched men [95,215]. Adiponectin has also been shown to be higher in females than in weight-matched males [216,217]. Yet, pre-menopausal women are known to be protected against CVDs, demonstrating the potent role of estrogen in protecting them from metabolic-associated CVDs. Thus, not only do sex hormones dictate adipose tissue distribution, but they can, as seen in pre-menopausal women, limit any negative effect that increased adipose tissue in the body can cause.

### 4.3 Liver pyruvate kinase

Pyruvate kinase (PK) is an essential enzyme in the glycolysis pathway, regulating cell metabolism and ATP production. It catalyzes the reaction, which irreversibly converts phosphoenolpyruvate and ADP to pyruvate and ATP [218,219]. There are four tissue-specific isozymes of PK in humans: PKL (liver), PKR (erythrocytes), PKM1 (heart, CNS, and muscle), and PKM2 (embryonic tissue, tumor cells) [220–222]. The PKLR gene encodes PKL and PKR, while the PKM gene encodes PKM1 and PKM2 via alternative splicing [221–224]. Lee et al. performed an integrative network analysis to identify shared biological pathways between gene targets, which could be beneficial in developing new therapeutic interventions for several liver diseases, including MASLD. The PKLR gene was identified as a potential target for effective treatment [224]. In a 2018 study, Krishnan et al. used an integrative genomics approach to pinpoint the PKLR gene as one of the key drivers for hepatic steatosis and MASLD development [225]. Since this study used only male mice, there was no data regarding the role of LPK in females. In 2021, Krishnan et al. used information from the previous study and data from human tissues and mouse models to determine sex differences in LPK effects on developing hepatic steatosis. In this study, LPK expression in both male mice and humans positively correlated with liver triglyceride levels, while there was no correlation in female tissue samples. LPK silencing improved metabolic phenotypes such as glucose tolerance and insulin sensitivity only in male mice [226]. This suggests that LPK has no role in developing MASLD in females. A 2020 study revealed that testosterone is dominant in regulating sexually dimorphic gene expression in the liver [227]. This supports the finding that LPK expression correlates with the severity of hepatic steatosis in male subjects. This finding also highlights the significant influence of sex hormones on gene expression between males and females and may explain further why men are more likely to be diagnosed with MASLD.

PKM2 has historically been connected to tissues with significant cellular proliferation, such as embryonic tissues and tumors [222,228]. However, upregulation of PKM2 expression has been seen in metabolic disorders such as T2D [229] and MASLD [230,231], as well as many cardiovascular diseases [232].

Obese individuals were found to have high serum PKM2 levels, which positively correlated with BMI before gastric bypass surgery. Post-surgical PKM2 decreased, but serum PKM2 was no longer associated with BMI [230]. Meoli et al. also gathered data from MASLD/NASH patients, revealing higher PKM2 serum levels and a twofold increase in inducible nitric oxide synthase (iNOS) gene expression levels in these patients. In 2018, Ouyang et al. proposed a novel role for digoxin in treating hepatic steatosis and inflammation [233]. Typically, digoxin is prescribed to improve cardiac function, but this study shows that the drug suppresses PKM2, which inhibits HIF-1a transcription, leading to decreased inflammation and damage to the liver [233]. It is to be noted that only male mice were used in this study, so the effect of digoxin on MASLD in females is not known.

Increased PKM2 expression has been linked to several cardiovascular diseases. In 2022, Doddapattar et al. found reduced inflammation and slowed progression of atherosclerotic lesions in male and female myeloid-specific PKM2 knockout mice [234]. A study using human heart samples found increased PKM2 expression in patients with heart failure [235]. Another study, however, demonstrated a possible cardioprotective role for PKM2 overexpression in pressure-overload-induced heart failure patients. By uncovering that PKM2 inhibits the RAC1 (rho family, small GTP binding protein) -MAPK (mitogen-activated protein kinase) signaling pathway, they found delayed progression of heart failure and cardiac hypertrophy [236]. Studies exploring the mechanisms linking PKM2 to CVDs are becoming more prevalent; however, the need for detailed studies regarding sex differences cannot be overlooked.

### 4.4 Ketone bodies

The liver produces ketone bodies during periods of caloric restriction, such as fasting [237], and several studies have documented its protective role against MASLD and cardiovascular dysfunction [238–240] (Figure 3). Ketogenesis in the liver eliminates up to two-thirds of the lipids entering the liver. B-hydroxybutyrate, the most abundant ketone body, protects the liver against steatosis by exerting anti-inflammatory, anti-oxidative, and anti-ER stress effects and inhibiting intrahepatic lipid deposition [239,241]. This suggests that disruption in the ketogenic pathway can strongly influence the development of hepatic steatosis [241]. Furthermore, ketone utilization benefits the heart by enhancing mitochondrial function, endothelial function, and vasodilation while reducing body weight, oxidative stress, cardiac remodeling, and inflammation [242]. Different ketone levels in males and females have been observed during fasting. The increase in ketone body levels associated with short-term fasting is greater in women than men [243]. It has also been reported that premenopausal females are protected against hepatic lipid accumulation due to the partition of fatty acids towards ketone body production rather than the influence of very LDL–triacylglycerol [244]. Animal studies from our lab have shown that low ketone levels in PPARα knockout male mice are associated with hepatic steatosis and cardiovascular dysfunction, suggesting that ketones might be involved in protection against MASLD-induced CVDs at least in males [31,245]. Animal studies using genetic modifications or pharmacological approaches have provided some mechanistic insight into the role of ketones in the development of cardiac and metabolic disease, and sex differences may play a role. In female mice, hepatocyte-specific knockout of D-3-Hydroxy-n-butyrate dehydrogenase (BDH1), an enzyme that catalyzes the last step of hepatic ketogenesis, provokes hepatic steatosis during fasting, suggesting the role of ketogenesis in lipid homeostasis in the liver [246]. On the other hand, cardiac BDH1 knockout male mice exhibit worse pathological cardiac remodeling following pressure overload/ischemic damage compared to controls [247]. Although some evidence suggests varied metabolic responses between males and females, which might be potentially due to hormonal differences and disparities in adipose tissue distribution, more studies need to be done on the potential sexual dimorphism in ketone body production during the development and progression of metabolic diseases and how this impacts the development of subsequent CVD.

### 4.5 Mitochondrial bioenergetics

Recent studies have demonstrated sexual dimorphism in mitochondrial bioenergetics, which is essential in energy metabolism [248,249]. Mitochondria from females exhibit upregulated antioxidant capacity, respiratory function, biogenesis, and lower reactive oxygen species production than in mitochondria isolated from males [250]. In female hepatocytes, mitochondria have higher protein content, greater respiratory rate, cardiolipin levels, and OXPHOS capacity than that observed from hepatocytes isolated from males [251]. In addition, cardiomyocyte mitochondria are differentially regulated between males and females. Like the hepatocytes, mitochondria isolated from female cardiomyocytes have a greater antioxidant capacity, produce less reactive oxygen species, and have greater calcium retention capacity, which might contribute to cardioprotection [250]. Scott et al. reported increased resistance to acute stress, inflammation, and oxidative stress in cardiac mitochondria isolated from females compared to males [248]. Since there are convincing reports on the role of mitochondrial function in the pathogenesis of MASLD and CVDs [252,253], these results taken together imply that superior mitochondrial function in females may contribute to cardiometabolic protection against MASLD and CVDs (Figure 3).

### 4.6 Insulin resistance

Insulin resistance is a critical factor in developing CVDs in patients with MASLD. Insulin resistance is central to the development and progression of MASLD, and it is an independent risk factor for CVDs [8]. In MASLD, insulin resistance increases fatty acid entry into the liver, which stimulates lipogenesis and VLDL overproduction, and this is a central driver in atherosclerosis that causes cardiac damage [253]. Insulin resistance causes metabolic alterations that drive CVDs. Such alterations include dyslipidemia, atherosclerosis, vascular stiffness and high blood pressure, inflammation, endothelial dysfunction, and mitochondrial dysfunction [254,255]. Sex-specific differences occur in the level of insulin sensitivity between men and women. During the premenopausal stage, women are generally more insulin-sensitive than men because of hormonal differences [256]. However, insulin sensitivity declines with the onset of the menopausal phase, and the risk of developing insulin resistance in menopausal women becomes similar to men, suggesting the critical role of female hormones in insulin sensitivity. In a study on postmenopausal women with coronary disease, estrogen replacement therapy reduced the incidence of diabetes by 35% [257]. In another study, estrogen replacement in postmenopausal women without diabetes reduced abdominal obesity, insulin resistance, new-onset diabetes, lipids, blood pressure, and adhesion molecules in women with diabetes [258].

It is also clear from animal studies that male rodents fed with a high-fat diet exhibit obesity associated with insulin resistance than females [259]. Estrogen contributes to insulin sensitivity by regulating insulin secretion and delivery, suppressing hepatic glucose production, improving glucose homeostasis in insulin-sensitive tissues, and suppressing inflammation and oxidative stress to protect against insulin resistance (241–243). Knockout of estrogen receptor α or aromatase, an enzyme that converts androgens into estrogens in mice, caused insulin resistance (244, 245). Furthermore, in ovariectomized high-fat diet-fed mice, estradiol replacement reduced insulin resistance by 50% (246). Taken together, females are protected from insulin resistance and its impact on MASLD-induced CVDs before menopause due to estrogen (Figure 3).

## 5. Conclusion

Research in the past years has revealed that sex differences influence the development of MASLD and, consequently, its complications of CVDs. Females display less severity in the progression of MASLD-induced CVD during their premenopausal phase compared to males of similar age. Apart from estrogens and androgens, other factors that contribute to sex-based differences include separate phases of reproductive life, ethnicity, age, lifestyle, genetic predispositions, differences in adipose tissue distribution, and the hormones they produce. However, several areas remain unclear. For example, the sex-based roles of various other genes identified in MASLD and their association with CVDs are yet to be investigated. Furthermore, the rapid loss of cardiovascular protection in females after menopause has been reported, but the mechanisms involved have not been fully elucidated.

Finally, understanding the sex-based mechanisms underlying how MASLD drives CVDs could help identify sex-specific therapeutic potential for patients. However, sex-based differences in mechanisms of MASLD-induced CVDs require further investigation.

## Data Availability

No new data were created for this review.
